# Phenotypic and functional alterations of pDCs in lupus-prone mice

**DOI:** 10.1038/srep20373

**Published:** 2016-02-16

**Authors:** Zhenyuan Zhou, Jianyang Ma, Chunyuan Xiao, Xiao Han, Rong Qiu, Yan Wang, Yingying Zhou, Li Wu, Xinfang Huang, Nan Shen

**Affiliations:** 1 Shanghai Institute of Rheumatology, Renji Hospital, School of Medicine, Shanghai Jiaotong University, Shanghai, China; 2 Institute of Health Sciences, Shanghai Institutes for Biological Sciences (SIBS) & Shanghai Jiao Tong University School of Medicine (SJTUSM), Chinese Academy of Sciences (CAS), Shanghai, China; 3 Division of Rheumatology and the Center for Autoimmune Genomics and Etiology (CAGE), Cincinnati Children’s Hospital Medical Center, Cincinnati, Ohio, United States of America; 4 Tsinghua-Peking Joint Center for Life Sciences, Tsinghua University School of Medicine, Beijing, China

## Abstract

Plasmacytoid dendritic cells (pDCs) were considered to be the major IFNα source in systemic lupus erythematosus (SLE) but their phenotype and function in different disease status have not been well studied. To study the function and phenotype of pDCs in lupus-prone mice we used 7 strains of lupus-prone mice including NZB/W F1, NZB, NZW, NZM2410, B6.NZM^*Sle1/2/3*^, MRL/*lpr* and BXSB/Mp mice and C57BL/6 as control mice. Increased spleen pDC numbers were found in most lupus mice compared to C57BL/6 mice. The IFNα-producing ability of BM pDCs was similar between lupus and C57BL/6 mice, whereas pDCs from the spleens of NZB/W F1 and NZB mice produced more IFNα than pDCs from the spleens of C57BL/6 mice. Furthermore, spleen pDCs from MRL-*lpr* and NZM2410 mice showed increased responses to *Tlr7* and *Tlr9,* respectively. As the disease progressed, IFN signature were evaluated in both BM and spleen pDC from lupus prone mice and the number of BM pDCs and their ability to produce IFNα gradually decreased in lupus-prone mice. In conclusion, pDC are activated alone with disease development and its phenotype and function differ among lupus-prone strains, and these differences may contribute to the development of lupus in these mice.

System lupus erythematosus (SLE) is the most common autoimmune disease in young women. The course of SLE varies greatly among individuals, ranging from mild to rapidly progressive and even fatal disease. Patients with SLE usually present with high interferon α (IFNα) levels in peripheral blood cells^1^. Moreover, patients with chronic hepatitis who receive IFNα therapy can develop lupus-like symptoms^2^. Recent studies have found that neutralizing IFNα can decrease the autoantibody titer and relieve clinical symptoms in both human SLE patients and lupus-prone mice^3^,^4^,^5^,^6^. Therefore, the abnormal activation of the IFNα pathway is considered one of the important mediators of SLE pathogenesis^7^,^8^.

New Zealand Black (NZB) × New Zealand White (NZW) F1 (NZB/W F1) mice represent the first strain developed to exhibit symptoms that mimic lupus, including high titers of autoantibodies and severe nephritis^9^. This strain has been widely used in studies of lupus pathogenesis. High IFN levels have also been detected in the NZB/W F1 strain and its sibling strains, such as NZB, New Zealand Mixed (NZM) 2328, NZM2410, B6.NZM^*Sle1/2/3*^and B6.*Nba2* mice^10^,^11^,^12^,^13^. Similarly, other lupus models, such as BXSB/Mp and pristine-induced lupus models, also include expression of high levels of IFN^14^,^15^. Although the role of IFNα in MRL-*lpr* mice is controversial, IFN inducible genes have been upregulated in response to disease progression in MRL-*lpr* mice^16^. The abnormal activation of the type I IFN pathway has been detected in both human SLE patients and lupus-prone mice, although the IFN source remains unclear.

Almost all immune cells can produce IFNα, and plasmacytoid dendritic cells (pDCs) are by far the most important producers of IFNα. pDCs selectively express high levels of *Tlr7* and *Tlr9* when stimulated with viral microbes, bacterial DNA or Tlr7/9 ligands^17^. Human pDCs consist of CD11c^-^ BDCA2^+^BDCA4^+^CD123^+^ cells^18^, whereas mouse pDCs consist of CD11c^int^mPDCA1^+^B220^+^ cells^19^,^20^, i.e., mPDCA1, which are also known as BST2 or CD317. mPDCA1 could be recognized by the antibody 120G8. Notably, DNA- or RNA-containing immune complexes (ICs) from SLE patients trigger pDCs from healthy donors to produce IFNα via the TLR9 or TLR7 pathways, which suggests that INFα is linked to SLE pathogenesis^21^,^22^,^23^. Lupus-prone mice, such as NZB, NZW and NZB/W F1 mice, exhibit higher spleen pDC numbers than C57BL/6 mice^24^,^25^. Moreover, NZB mice show increased IFNα secretion following *in vivo* CpG injection^26^. Hence, we hypothesize that pDCs may represent an important IFNα source in both SLE patients and lupus-prone mice. The main function of pDCs is the efficient production of IFNα, which has become the focus of intense investigation^18^,^27^. Unfortunately, the availability of human pDCs is limited because pDCs account for only 0.1% of human PBMCs. Therefore, here, we studied pDCs derived from lupus-prone mice to illuminate the pathogenesis underlying SLE.

The aim of this study was to analyze the pDC phenotype and its IFNα-producing ability by following *Tlr7* and *Tlr9* stimulation in different lupus-prone mouse strains. Here, we studied 7 lupus strains and found increased pDC cell counts and function in the NZB/W F1, NZB, NZM2410 and MRL-*lpr* strains. In the advanced lupus stage, the number and function of pDCs changed according to the development of the disease.

## Results

### NZB/W F1, NZB and MRL-*lpr* mice had larger pDC counts

**Figure 1:**
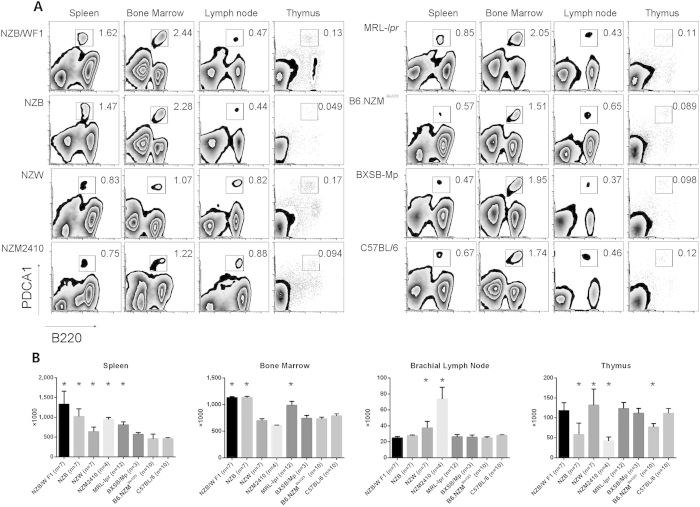
Numbers of pDCs in different lymphoid organs of different strains. Analysis of pDCs from different lymphoid organs in 6-week-old lupus-prone mice (8-week NZB mice). C57BL/6 mice were used as the control strain. LN-pDCs were isolated from 1 brachial LN, and BM pDCs were isolated from 2 tibias and 2 femurs in each mouse. The total pDC numbers were calculated by multiplying the organ pDC percentage by the total cell numbers. A: pDC percentages in different organs among different strains. B: pDC numbers among different organs. *p < 0.05 compared with the C57BL/6 strain.

We first hypothesized that the increased numbers of pDCs were responsible for the high IFN expression in lupus-prone mice. Here, we used only female mice for the experiments, for all strains except BXSB/Mp. The total cell numbers of different organs are given in Supplementary Table 1. The spleen pDC numbers were the highest in NZB/W F1 and NZB mice among all tested strains (Fig. 1A), followed by NZW, NZM2410 and MRL-*lpr*, whereas the numbers in B6.NZM^*Sle1/2/3*^and BXSB mice were not significantly different. Moreover, the (bone marrow) BM was also found to contain large numbers of pDCs in mice. Specifically, higher BM pDC levels were found in NZB, NZB/W F1 and MRL-*lpr* mice (Fig. 1B). The numbers of BM pDCs in NZW, NZM2410 and B6.NZM^*Sle1/2/3*^mice and male BXSB/Mp mice compared to that in C57BL/6 mice were not significantly different. Both the lymph nodes (LNs) and thymus glands contained fewer pDCs, suggesting that the LNs and thymus may contribute relatively little to the high IFN expression (Fig. 1C,D). However, the increased numbers of pDCs in the NZB/W F1, NZB and MRL-*lpr* strains suggested that pDCs may contribute to the high IFN expression. The IFN expression in the B6.NZM^*Sle1/2/3*^and BXSB/Mp strains was attributed to high pDC function, which prompted further investigation.

### BM pDCs produced higher levels of IFNα than pDCs from other lymphoid organs

**Figure 2:**
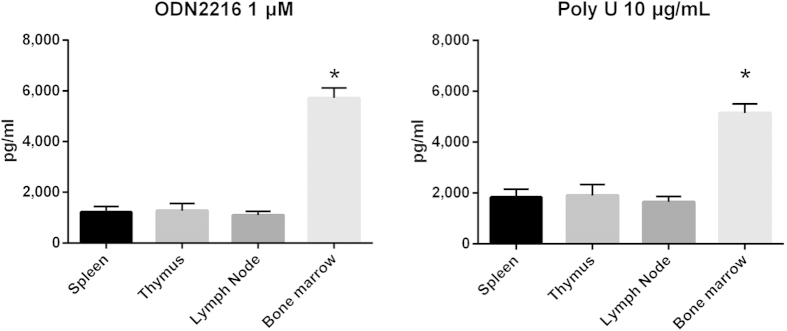
IFNα production by pDCs in different organs of C57BL/6 mice. IFNα produced by pDCs in different organs (BM, spleen, LNs, thymus) from C57BL/6 mice that were treated with Tlr ligands (ODN2216 for *Tlr9*, Poly U for *Tlr7*) for 48 h. Each well contained 50k cells. Cells from the inguinal, axillary, brachial and cervical superficial LNs were pooled together for LN-pDC isolation. *p < 0.05 compared with spleen pDCs

Previous studies have indicated that the ability of pDCs from diverse lymphoid organs to produce IFNα differed in C57BL/6 mice^28^,^29^. Therefore, we first compared the abilities of pDCs from the spleen, BM, LN and thymus of C57BL/6 mice to produce IFNα to assess the necessity of comparing pDC function separately in various lupus-prone mouse strains. The results suggested that BM pDCs could produce far more IFNα upon stimulation with both *Tlr7* and *Tlr9* than spleen pDCs, whereas the IFNα-producing abilities of thymus and LN pDCs were similar to that of spleen pDCs (Fig. 2). Thus, spleen and BM pDCs were selected for subsequent studies.

### Strong IFNα producing abilities of spleen pDCs from NZB/W F1, NZB, NZM2410 and MRL-*lpr* mice upon *Tlr7* or *Tlr9* stimulation

**Figure 3:**
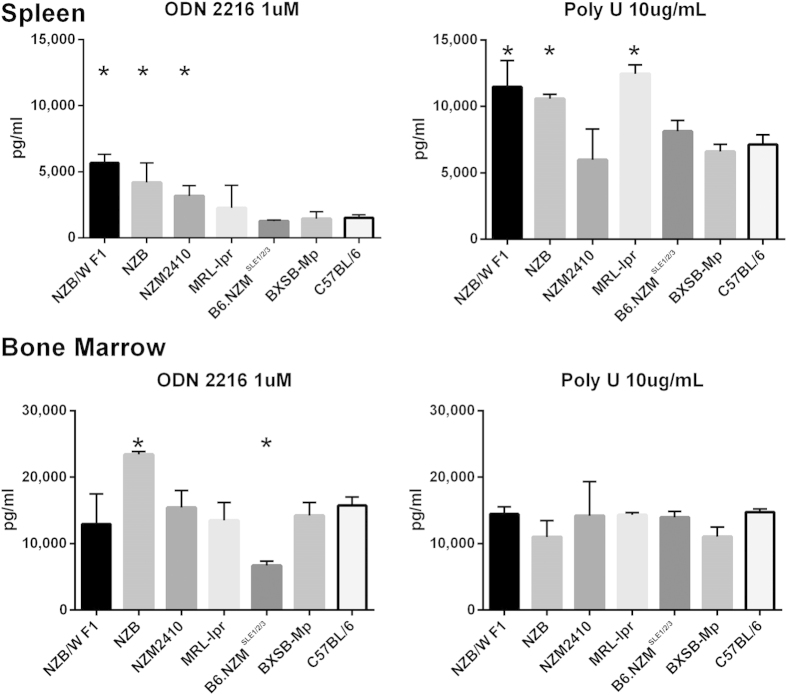
IFNα produced by spleen and BM pDCs from lupus-prone mice. Spleen and BM pDCs from different strains were stimulated for 48 h with TLR ligands. Each well contained 100 k cells. The figure shows the results of one of three independent experiments. *p < 0.05 compared with C57BL/6 mice.

The strong IFNα-producing abilities of pDCs from lupus-prone mice are also likely to contribute to the mechanism underlying the high IFN expression level. A comparison of the IFNα levels in response to stimulation with both *Tlr9* and *Tlr7* was necessary because either DNA- or RNA-containing ICs from lupus patients may stimulate pDCs to produce IFNα^21^,^22^,^30^. Here, we used ODN2216 and poly U as *Tlr9* and *Tlr7* ligands, respectively. Upon ODN2216 stimulation, spleen pDCs from NZB/W F1, NZB and NZM2410 produced higher levels of IFNα than spleen pDCs from C57BL/6 mice. BM pDCs from NZB and B6.NZM^*Sle1/2/3*^mice produced higher and lower levels of IFNα, respectively, than BM pDCs from C57BL/6 mice. In contrast, the production of IFNα by BM pDCs from other strains did not significantly differ from that of BM pDCs from C57BL/6 mice. The poly U stimulation results showed that spleen pDCs from NZB/W F1 and NZB mice also produced higher levels of IFNα. However, strikingly, spleen pDCs from MRL-*lpr* mice produced higher IFNα levels than those from control mice. Upon poly U stimulation, the BM pDCs from the various lupus strains did not differ (Fig. 3). Together, these results suggest that the overactivity of pDCs in some lupus strains may result in high IFN expression.

### pDCs from NZB and NZB/W F1 mice had higher survival ratios in *in vitro* stimulation

**Figure 4:**
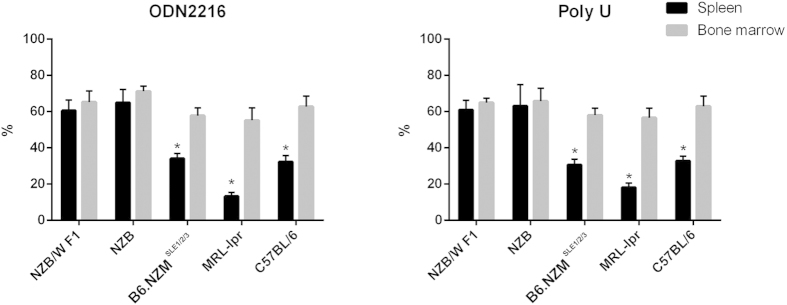
pDC survival rate after 24 h of stimulation with *Tlr7* or *Tlr9 in vitro* Spleen and BM pDCs from six-week-old mice were isolated and stimulated according to the protocols mentioned above. Previous studies already demonstrated that almost all pDCs die within 48h. Therefore, we compared the survival rates at 24 h. The pDC survival rate at 24 h in 5 tested strains. BM pDCs exhibited higher survival rates in B6.NZM^*Sle1/2/3*^, MRL/*lpr* and C57BL/6 strains in response to both ODN2216 and poly U stimulation. The survival rates of BM and spleen pDCs did not significantly differ in NZB and NZB/W F1 mice.

Zhan *et al.* have found that lower pDC death rates are linked to high levels of IFNα production *in vitro*^25^. To identify the reasons underlying the high IFNα production by BM pDCs and the strain differences in the pDC IFNα-producing ability, we analyzed the pDC survival rates in response to *Tlr7* or *Tlr9* stimulation *in vitro*. Previous studies have found that all pDCs died within 48h *in vitro*. Therefore, we collected the cells after 24 h and calculated the number of surviving pDCs. All BM pDCs showed higher survival rates than spleen pDCs after 24 h *in vitro*. Among the various strains, both spleen and BM pDCs from NZB and NZB/W F1 had the highest surviving cell proportions, regardless of the stimulator. Although pDCs from MRL-lpr mice produced higher levels of IFNα in response to *Tlr7* stimulation, their pDC survival rates were the lowest (Fig. 4), which suggested that in addition to increased pDC survival, other mechanisms may affect the IFNα-producing ability.

### BM pDCs exhausted in advanced lupus stage

Studies of the phenotype and function of pDCs in the advanced stages of lupus have been limited^31^. Therefore, we sought to determine how pDCs behave in the advanced stage of lupus in mouse models. We divided all lupus-prone mice into 3 different groups as follows: 6-week-old (or 8-week-old for NZB, owing to its physical retardation) mice were regarded as the pre-lupus stage, at which point neither proteinuria nor autoantibody is detectable. Here, we defined ANA titers “1:100 < ANA < 1:320” and proteinuria “+ ≤ protein ≤ 2+” as cut-off values for the early lupus stage, whereas ANA ≥ 1:320 or urine protein ≥ 3+ were used to define the advanced stage. To date, few studies have focused on the change in the total cell numbers in the BM of lupus mice. Our data showed that the total BM cell numbers were slightly increased in NZB/W F1 mice, but this increase was not observed in MRL-*lpr* and B6.NZM^*Sle1/2/3*^ mice (Table 1). The BM pDC numbers significantly decreased as the disease progressed in all 3 tested strains (see Table 2). Elderly C57BL/6 mice did not exhibit changes in pDC numbers in either the spleen or the BM.

Disease-induced pDC migration may decrease the number of pDCs in the BM. We also measured the number of pDCs in the peripheral organs, including the spleen, LN and kidney. NZB/W F1 mice exhibited slightly enlarged spleens, results corroborating the findings of a previous study^32^. In addition, MRL-*lpr* mice exhibited marked spleen enlargement, and the total number of spleen cells was up to 20-fold higher in the advanced stage lupus mice than in pre-lupus mice. Furthermore, the total spleen cell population of B6.NZM^*Sle1/2/3*^mice was up to 4–6 times larger in the advanced stage than in the pre-lupus stage (see Table 1). The absolute spleen pDC numbers were higher in MRL-*lpr* and B6.NZM^*sle1/2/3*^ mice than in NZB/W F1 mice (Table 2).

Moreover, lymphomegaly is common in lupus mouse models, and all tested strains exhibited various degrees of LN enlargement. Specifically, the LN in MRL-*lpr* strains was about 10 times larger in mice with advanced disease than in pre-lupus mice. However, the number of LN pDCs showed limited increases in the NZB/WF1 and B6.NZM^*Sle1/2/3*^strains (Supplementary Table 2). pDC infiltration in organs has also been reported to decrease the number of BM pDCs. The kidney is one of the most common organs affected by lupus. Fiore *et al.* have found BDCA4-positive cells in type III and type IV lupus nephritis renal tissue^33^, but related studies in mouse models have not been reported. The total renal pDC numbers were calculated on the basis of a FACS analysis. However, pDC infiltration in the kidney was not detectable in the pre-lupus stage, whereas the renal pDC numbers were slightly increased in response to disease development in all tested strains. Although the total renal pDC numbers were very low and almost undetectable, the total number of renal B220+ cells was significantly increased (Supplementary Fig. 3), which implied that pDCs expressing B220+ did not significantly infiltrate the kidneys of lupus-prone mice. At the advanced lupus stage, the number of renal pDCs greatly declined, which may have been the result of tissue fibrosis (Supplementary Table 2 & Supplementary 3).

Based on these results, the increased total pDC numbers in peripheral organs were not sufficient to compensate for the decreased pDC numbers in the BM. In fact, the lower number of BM pDCs may be caused by disease progression instead of pDC migration.

### Decreased IFNα-producing ability of BM pDCs upon ODN2216 simulation in advanced-stage lupus

**Figure 5:**
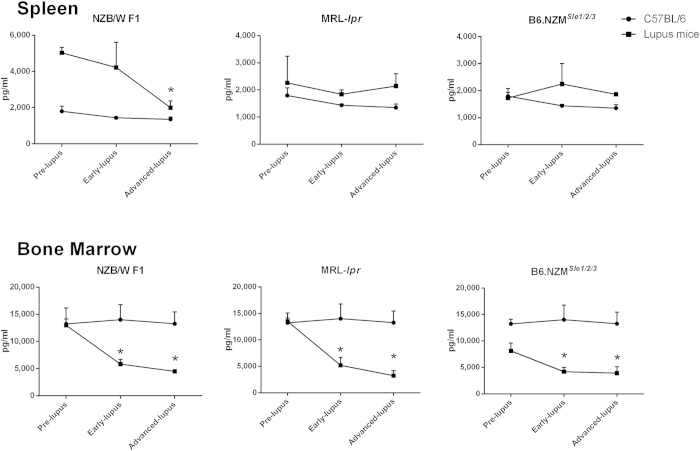
IFNα-producing abilities of pDCs from different disease stages. Only spleen pDCs from NZBW F1 mice exhibited decreases in IFNα production upon ODN2216 stimulation in the advanced lupus stage. Decreases in the IFNα-producing abilities of BM pDCs were observed in all 3 tested strains during the advanced lupus stage. *p < 0.05 compared with the pre-lupus stage

The effect of disease status on the IFNα-producing ability has not yet been studied. Therefore, we collected spleen and BM pDCs from NZB/W F1, B6.NZM^*Sle1/2/3*^ and MRL-*lpr* mice in different disease stages and then challenged them with ODN2216. BM-pDCs from NZB/W F1 mice lost their responses to stimulation in the pre- and advanced lupus stages. Similarly, spleen pDCs from F1 mice in the advanced lupus stages also revealed decreased responses (Fig. 5). The loss of IFNα production was also observed in BM pDCs from MRL-*lpr* and B6.NZM^*Sle1/2/3*^mice but not in their spleen pDCs. The pDC function of age-matched C57BL/6 mice remained unchanged.

### pDC was activated in lupus-prone mice in the advanced lupus stage

**Figure 6:**
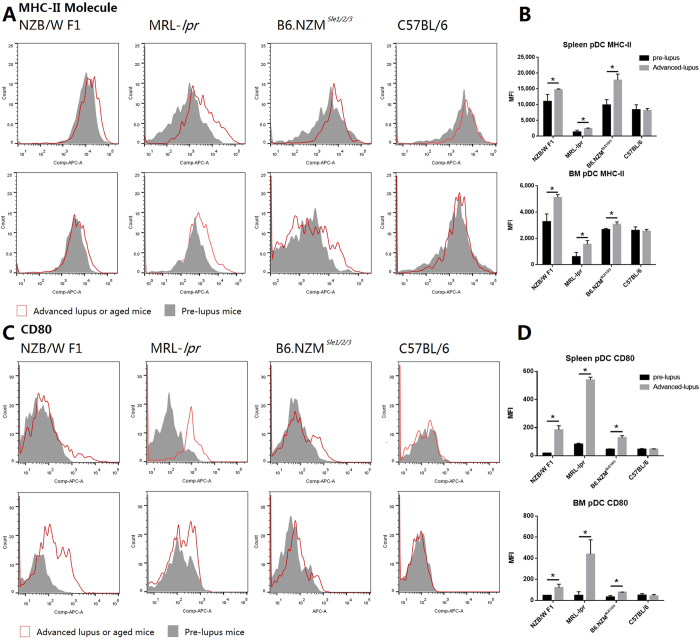
Increased MHC-II molecule and CD80 expression in pDCs from advanced lupus mice In total, 1 × 10^7^ cells obtained from the spleen or BM were stained with CD11c, mPDCA-1 and MHC-II or CD80. CD11c^+^mPDCA-1^+^ cells were gated, and we compared the MHC-II or CD80 levels. (**A**) MHC-II molecule expression levels in spleen and BM pDCs at the early and advanced lupus stages. The upper row shows spleen pDCs, and the lower row shows BM pDCs. (**B**) Statistical analysis of the MFI of the MHC-II molecule. *p < 0.05 compared with the pre-lupus stage. (**C**) CD80 expression level in pre-lupus and advanced lupus spleen and BM pDCs. Top rows of A and C are spleen pDCs, and bottom rows are BM pDCs. D. Statistical analysis of the MFI of CD80. *p < 0.05 compared with the pre-lupus stage

**Figure 7:**
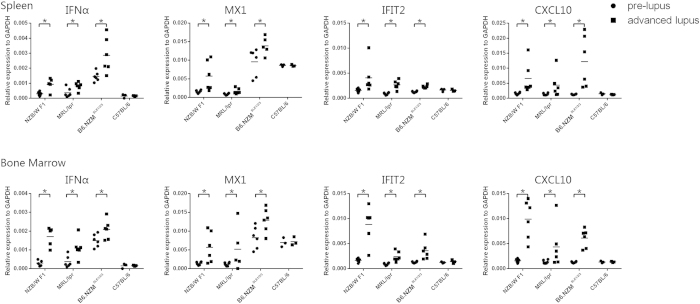
IFNα- and IFN-inducible gene expression levels by disease stage. Here, we used 3 different lupus strains—NZB/W F1, MRL-*lpr* and B6.NZMSle1/2/3—to test the IFNα gene expression and IFN-inducible gene expression. The pre-lupus and advanced lupus stages are described above. Six-week- and 6-month-old C57BL/6 mice served as controls. In the 3 tested lupus strains, both the spleen and BM IFNα expression levels were elevated. Three IFN-inducible genes, MX1, IFIT2 and CXCL10, were also universally upregulated in both the spleen and BM pDCs in all 3 tested strains. Elderly C57BL/6 mice did not show increases in IFNα expression and IFN-inducible gene expression in the spleen or BM pDCs.

The upregulation of both MHC-II and CD80 in pDCs is a hallmark of pDC activation. The levels of both MHC-II and CD80 in spleen and BM pDCS in all 3 tested strains were significantly upregulated in the advanced lupus stage compared with the pre-lupus stage, whereas this difference was not observed in control C57BL/6 mice (Fig. 6). The IFN inducible genes is considered to be another marker of pDC activation. To date, more than 30 genes have been reported to be induced by type I interferon. Because only limited numbers of pDCs can be obtained from individual mice, we analyzed the pDC activation status of 3 genes: MX1, IFIT2 and CXCL10, we also added pan-IFNα genes to further test the activation status. In all 3 tested strains, both IFNα genes and IFN inducible genes expression levels were elevated in both the spleen and BM pDCs in the advanced lupus stage but not in elderly C57BL/6 controls (Fig. 7).

Taken together, these data indicate that pDCs are activated at the advanced lupus stage in lupus mouse models.

## Discussion

The underlying pathogenesis of SLE is elusive and complex because of a wide range of potential disease mechanisms among individuals. To mimic these different mechanisms, we used 7 different lupus-prone mouse strains^34^,^35^. NZB/W F1 mice, the first model lupus-prone strain, presented with a high titer of autoantibodies and severe nephritis. NZB/W F1 mice were generated by mating female NZB mice and male NZW mice. Both NZB and NZW mice develop nephritis and autoantibodies at the later stages of the disease, but unlike NZB/W F1 mice, lupus symptoms in these 2 strains are often mild. The life span of NZW mice is similar to that of C57BL/6 mice, whereas NZB mice have a shorter life span. In NZB mice, B cells are unusually mature, hyperactivated and resistant to apoptosis. CD4^+^ T cells also contribute to the disease of NZB mice because of the types of MHC-II molecules expressed by these mice. Naturally, hemolytic anemia instead of lupus is a major cause of death in NZB mice^26^. NZB/W F1 is the only heterozygous strain that inherits disease-related gene loci from both NZW and NZB mice and develops severe lupus symptoms. A previous study has observed polyclonal B cell activation with the help of both α/β T and γδ T cells in NZB/W F1 mice. The functional impairment of regulatory cells, including CD4^**+**^CD25^**+**^, CD8^**+**^, NK T, and B-1 B cells, has been found in NZB/W F1 mice^26^. The NZM2410 strain, which was generated by repeated backcross mating of NZB/W F1 mice to NZW mice, was discovered to have *Sle1*, *Sle 2* and *Sle 3* lupus-related gene loci. These 3 disease related loci also exist in NZW mice. Each of these three gene loci was able to induce autoimmunity in C56BL/7 mice and B6.NZM^*Sle1/2/3*^ mice that bear all three loci; these mice exhibited hyperproliferative and hyperactive T and B cells, which caused severe nephritis and high titers of autoantibodies^36^. Although NZM2410 and B6.NZM^*Sle1/2/3*^ mice have the same disease-related gene loci, they present with different clinical symptoms. Previous studies have found that NZM2410 mice develop glomerulosclerosis at the early stage of nephritis, whereas other mice develop diffuse proliferative nephritis^26^. In our work, B6.NZM^*Sle1/2/3*^ mice also presented with chondritis, as evidenced by ear collapse, conjunctivitis and dermatitis, which might be the result of different immune microenvironments. MRL/Mp mice, which were generated by backcrossing 4 different strains, including LG, AKR, C3H/Di and C57BL/6, exhibited dermatitis, lower titers of autoantibodies and mild nephritis in later life^26^. MRL/Mp mice with the *lpr* mutation developed Fas deficiency, resulting in higher titers of autoantibodies and severe nephritis. Autoreactive T and B cells that fail to undergo apoptosis are considered to be characteristic of pathogenesis in the MRL-*lpr* strain^26^. The BXSB/Mp strain, which was generated by backcrossing SB/Le mice and C57BL/6 mice^37^, carries the *Yaa* mutation originating from the SB/Le strain. This mutation results in the insertion of 17 genes from the X chromosome into the Y chromosome. Among the 17 genes in the *Yaa* mutation, Tlr7 is considered to be the major cause of lupus^38^,^39^. Because gene imprinting does not silence the Y chromosome, all male mice carrying this mutation express high levels of *Tlr7* in B cells and pDCs^38^. Intriguingly, C57BL/6 mice with only the *Yaa* mutation do not develop clinical lupus, but the introduction of the *Sle1* locus into B6.*Yaa* mice (B6.NZM^*Sle1*^*.Yaa* strain) results in severe lupus symptoms similar to those exhibited by BXSB/Mp mice. The *Sle1* locus induces the development of autoreactive T and B cells in C57BL/6 mice, and a Bxs1 locus exhibiting functions similar to those of the *Sle1* locus has been discovered in the BXSB/Mp strain^40^. Therefore, *Yaa* mutation could cause high levels of *Tlr7* expression in pDC and further facilitate the in autoimmune pathogenesis caused by suspected lupus loci through amplifying the effects of *Tlr7*-mediated pathogenic pathway^41^,^42^.

As described previously, the source of IFN in SLE patients remains unknown. Lupus-prone mice universally present high IFN signature, thus providing excellent animal models to investigate this issue. Recent studies have found that pDC depletion relieves lupus symptoms and downregulates IFN expression in both B6.*Nba2* and BXSB-DTR mice^43^,^44^. Our study herein found that pDC activation markers, including MHC-II molecules, CD80, IFNα and IFN signature, were upregulated in lupus-prone mice at the advanced lupus stage, thereby indicating that pDCs were activated and released IFNα in lupus-prone mice. Despite the lack of human data in our study, previous studies have found that RNA- and DNA-containing ICs from SLE patients stimulates pDCs from healthy donors, thus producing IFNα *in vitro*. These findings, together with our results, indicate that pDCs constitute an important IFNα source in SLE patients.

Despite of the large variation in pDC numbers and function among different mouse strains, all lupus-prone mice could develop disease. In our study, NZB mice with mild lupus symptom showed the highest pDC number and strong IFNα producing ability, while B6.NZM^*Sle1/2/3*^and BXSB/Mp mice with much more severe lupus symptoms than NZB mice showed similar pDC function and phenotype to C57BL/6 mice. These findings indicate that increased pDC number and enhanced cytokine production by pDC are not directly linked to the development and/or severity of the lupus in mouse models. Since pDC itself is one of the strongest IFNα-producing cell in mouse, thus the activation of pDCs and their cytokine production are expected to influence the disease progression. Indeed, increased pDC number and/or enhanced pDC function in some lupus models would enhance the toxic effect of IFNα like NZB/W F1 mice.

In this study, spleen pDCs from NZM2410 and MRL-*lpr* mice showed inconsistent selective responses to *Tlr9* and *Tlr7* stimulation, but the detailed mechanisms underlying this response remain unclear. On the basis of previous reports, we speculate that this phenomenon may be related to differences in SLE pathogenesis in these models. NZM2410 mice do not generate anti-RNP antibodies^26^, which suggests that this strain cannot produce RNA-containing ICs. Thus it is likely that pDC avtication in the NZM2410 strain *in vivo* relies on sensor DNA containing IC through *Tlr9* pathway, but not the *Tlr7* pathway, which requires the presence of RNA-containing ICs. Conversely, MRL-*lpr* mice produced both anti-dsDNA antibodies and anti-RNP antibodies, suggesting that this strain has both DNA- and RNA-containing ICs. Previous studies have found that *Tlr9* knockout MRL-*lpr* mice do not exhibit ameliorated clinical symptoms of disease, whereas *Tlr7*-deficient MRL-lpr mice exhibit decreased IFN signature and the remission of clinical symptoms^45^,^46^,^47^,^48^,^49^. This finding implies that pDC activation in the MRL-*lpr* strain may depend on selective *Tlr7* pathways that more actively interact with RNA-containing ICs to release IFNα. The reasons underlying this phenomenon remain unknown, but the genetic background may contribute to this effect. Nevertheless, this inference requires confirmation, and further studies are required to determine whether this phenomenon also exists in human patients.

Additionally, the number of BM pDCs, which produce much higher levels of IFNα than spleen pDCs, was significantly decreased in the advanced stage of lupus. This depletion may result from immune regulation at the advanced lupus stage, at which point a wide range of immune cells are activated, thus resulting in release of large amounts of cytokines, especially type I interferon. Because the immune environment may substantially change throughout disease progression, pDC proliferation might be suppressed by negative immune-regulation mechanisms, which suggests that BM pDC depletion results from a comprehensive immunoreaction negative feedback loop. Moreover, previous studies have demonstrated that activated pDCs do not respond to a second stimulus^17^, our date found pDC activation in lupus-prone mice in advanced disease stage, therefore pDCs isolated from mice in advanced lupus stage have reduced IFNα producing ability upon stimulation *in vitro*.

One of the important functions of pDC is to present antigen and elicit subsequent T cell activation and this function maybe enhanced in lupus-prone mice. OT-I and OT-II mice are widely used tools to investigate the antigen presenting function of antigen presenting cells (APCs) to prime CD8+ T cell and CD4+ T cell respectively. Of note, these mice were generated in C57BL/6 background and present H-2b haplotype. The lupus-prone mice used in current study have different genetic background and H-1 haplotypes as follows: NZB/W F1 (H-2^d/z^), NZB (H-2^d/d^), NZW (H-2^z/z^), NZM2410 (unknown, possibly H-2^z/z^), MRL-*lpr* (H-2^k/k^), B6.NZM^*Sle1/2/3*^(H-2^b/b^) and BXSB/Mp (H-2^b/b^). To test the antigen-specific T cell activation, APCs are required to present the same H-2 molecules as T cells. As mentioned above, abnormal T cells were also found in most lupus-prone mice. Therefore current methods and technologies cannot give the answer that APC function is enhanced in lupus-prone mice.

In summary, our data indicate that pDCs are activated in lupus-prone mice. We also found abnormal pDC phenotypes and function in some lupus-prone mice, which may increase the IFN expression in advanced disease. Our findings provide information that may guide future studies of SLE patients, but they will require validation in humans.

## Materials and Methods

### Ethics approval

All the animal experiments in this study were approved by the Ethic Committee of Renji Hospital, School of Medicine, Shanghai Jiao Tong University, and were performed in accordance with the guidelines for animal experimentation of Shanghai Jiao Tong University.

### Mice

NZB, NZW, NZM2410, MRL-*lpr*, MRL/Mp, B6.NZM^*Sle1/2/3*^, C57BL/6, BXSB/Mp, and BXSB.B6-*Yaa* mice were purchased from Jackson Laboratories (Bar Harbor, ME, USA). All animals were bred at the animal facility of Renji Hospital, School of Medicine, Shanghai Jiao Tong University. The NZB/W F1 strain was generated by breeding male NZW and female NZB mice. We used the commonly used C57BL/6 mice as a reference strain, and all statistical analyses were based on comparisons with C57BL/6 mice in this study unless otherwise indicated.

### Mouse pDC isolation and phenotypic analysis

pDCs were isolated from the spleen, LNs, thymus and BM of individual mice as described elsewhere in detail^50^. Briefly, the organs were minced and digested with DNase/collagenase solution. The BM cells were flushed from 2 femurs and 2 tibias per mouse with PBS. To isolate the pDCs, the total cells obtained from the spleen, LNs, thymus and BM were resuspended in Nycodenz (Nycomed Pharma AS, Oslo, Norway) medium (1.077, 1.082, 1.076 and 1.080 g/mL, respectively) and centrifuged at 1700 x g for 10 min. Cells in the light density fraction were then harvested and labeled with CD11c, mPDCA-1, B220 and aqua for sorting. The finial pDC purity exceeded 95% (Supplementary Fig. 1). PerCP.Cy5.5-, PE- and APC-labeled isotypes were introduced as negative controls. To analyze the pDC numbers and phenotypes, 1 × 10^7^ cells from organs were used and labeled with CD11c, mPDCA-1, B220, CD80 and MHC-II. The pDC gating strategies are provided in Supplementary 3A. The pDC numbers were calculated by multiplying the total organ cell number by the percentage of CD11c^int^mPDCA-1^+^B220^+^ cells. The mean fluorescence intensities (MFIs) of MHC-II and CD80 of pDC were calculated by FlowJo software (Treestar Inc., Ashland, OR, USA). The living cell rates were detected by using Annexin V and PI staining. 100K of pDC were stimulated with ODN2216 or Poly U for 24 h and then performed Annexin V and PI staining follow the protocol from the manufacture (BD Pharmagen, San Jose, CA, USA). The gating strategy could be found in supplementary fig. 2B. Anti-mouse CD11c, mPDCA-1, B220, CD80, MHC-II and isotypes were purchased from ebioscience (San Diego, CA, USA). Aqua was purchased from ThermoFisher Scientific (Waltham, MA, USA).

### *In vitro Tlr9* and *Tlr7* stimulations

Isolated spleen or BM pDCs (100k) were resuspended in 100 μL of complete 1640 medium and then stimulated in a 96-well U-bottom plate with 1-μM ODN2216 or 10-μg/mL poly U (Invivogen, San Diego, CA, USA) supplemented with Lipofectamine 2000 (ThermoFisher Scientific) (1.5 μL: 1000 μL) for 48 h. The supernatants were collected and stored at −80 °C for enzyme-linked immunosorbent assay (ELISA). Each group consisted of at least 3 mice, and each experiment was repeated at least 3 times.

### ELISA

A mouse IFNα ELISA (eBioscience) was performed according to the manufacturer’s protocol. We used a QUNTA Lite (Inova Diagnostic, San Diego, CA, USA) kit by switching the 2nd antibody to FITC-conjugated anti-mouse IgG (Santa Cruz Biotechnology, Dallas, Texas, USA).

### Quantitative polymerase chain reaction (qPCR) analysis

The pDCs were lysed in TRIzol (ThermoFisher Scientific) and then maintained at −80 °C for RNA extraction. The total RNA was extracted following the manufacturer’s protocol. cDNA was synthesized by using TaqMan Reverse Transcription Reagents (ThermoFisher Scientific), and 1:100-diluted cDNA was used to perform qPCR using SYBR® Premix Ex Taq (TAKARA BIO INC., Shiga, Japan). The primer information is provided in the supplementary material. Relative expression levels were calculated by using the delta-delta CT method. A P value < 0.05 was considered to indicate a significant difference.

### Statistics

The data were analyzed with Student’s t test by using GRAPHPAD PRISM V6 (Graphpad Software, San Diego, CA, USA).

## Additional Information

**How to cite this article**: Zhou, Z. *et al.* Phenotypic and functional alterations of pDCs in lupus-prone mice. *Sci. Rep.* **6**, 20373; doi: 10.1038/srep20373 (2016).

## Supplementary Material

Supplementary Information

## Figures and Tables

**Table 1:** Total cell numbers in spleen and BM (2 femurs and tibias) in lupus-prone mice at different disease stages.

Strains	Spleen (Mean ± SD) × 10^6^			BM (Mean ± SD) × 10^6^		
	Pre-lupus	Early lupus	Advanced lupus	Pre-lupus	Early lupus	Advanced lupus
NZB/W F1	109.9 ± 8.1	123.8 ± 6.9*	155.8 ± 24.8*	42.1 ± 2.1	48.3 ± 3.5*	46.8 ± 3.6*
MRL-*lpr*	106.7 ± 1.5	345.0 ± 22.6*	2175.0 ± 473.8*	43.3 ± 4.0	45.5 ± 5.2	47.6 ± 7.2
B6.NZM^*Sle1/2/3*^	109.2 ± 4.0	312.7 ± 72.7*	763.6 ± 141.9*	43.0 ± 6.3	44.7 ± 7.1	39.5 ± 6.8
C57BL/6	93.4 ± 5.1	95.8 ± 6.2	101.9 ± 6.4	47.3 ± 6.1	47.9 ± 5.5	46.4 ± 7.1

Age-matched C57BL/6 were used as a control. *P < 0.05 compared with the pre-lupus stage.

**Table 2:** The change in the pDC numbers in the spleen and BM as a function of disease stage.

Strains	Spleen pDC (Mean ± SD) × 10^6^			BM pDC (Mean ± SD) × 10^6^		
	Pre-lupus	Early lupus	Advanced lupus	Pre-lupus	Early lupus	Advanced lupus
NZB/W F1	1.502 ± 0.45	1.158 ± 0.75*	1.498 ± 0.28	1.07 ± 0.12	0.476 ± 0.05*	0.432 ± 0.10*
MRL-*lpr*	0.841 ± 0.11	1.051 ± 0.75	3.238 ± 1.46*	0.551 ± 0.07	0.349 ± 0.09*	0.227 ± 0.05*
B6.NZM^*sle1/2/3*^	0.460 ± 0.12	0.720 ± 0.14	1.294 ± 0.40*	0.571 ± 0.10	0.482 ± 0.13*	0.391 ± 0.05*
C57BL/6	0.455 ± 0.03	0.452 ± 0.04	0.427 ± 0.05	0.773 ± 0.08	0.662 ± 0.04	0.653 ± 0.17

Age-matched C57BL/6 were used as a control. *P < 0.05 compared with the pre-lupus stage.

## References

[b1] Tang J. *et al.* Increased expression of the type I interferon-inducible gene, lymphocyte antigen 6 complex locus E, in peripheral blood cells is predictive of lupus activity in a large cohort of Chinese lupus patients. Lupus 17, 805–813 (2008). 18755862 10.1177/0961203308089694

[b2] Sanchez Roman J., Castillo Palma M. J., Garcia Diaz E. & Ferrer Ordinez J. A. [Systemic lupus erythematosus induced by recombinant alpha interferon treatment]. Medicina clinica 102, 198 (1994). 8127174

[b3] Higgs B. W. *et al.* A phase 1b clinical trial evaluating sifalimumab, an anti-IFN-alpha monoclonal antibody, shows target neutralisation of a type I IFN signature in blood of dermatomyositis and polymyositis patients. Ann Rheum Dis 73, 256–262 (2014). 23434567 10.1136/annrheumdis-2012-202794 PMC3888620

[b4] Zagury D. *et al.* IFNalpha kinoid vaccine-induced neutralizing antibodies prevent clinical manifestations in a lupus flare murine model. Proc Natl Acad Sci USA 106, 5294–5299 (2009). 19279210 10.1073/pnas.0900615106 PMC2654395

[b5] Mathian A. *et al.* Active immunisation of human interferon alpha transgenic mice with a human interferon alpha Kinoid induces antibodies that neutralise interferon alpha in sera from patients with systemic lupus erythematosus. Ann Rheum Dis 70, 1138–1143 (2011). 21389044 10.1136/ard.2010.141101

[b6] McBride J. M. *et al.* Safety and pharmacodynamics of rontalizumab in patients with systemic lupus erythematosus: results of a phase I, placebo-controlled, double-blind, dose-escalation study. Arthritis Rheum 64, 3666–3676 (2012). 22833362 10.1002/art.34632

[b7] Hooks J. J. *et al.* Immune interferon in the circulation of patients with autoimmune disease. N Engl J Med 301, 5–8 (1979). 449915 10.1056/NEJM197907053010102

[b8] Preble O. T., Black R. J., Friedman R. M., Klippel J. H. & Vilcek J. Systemic lupus erythematosus: presence in human serum of an unusual acid-labile leukocyte interferon. Science 216, 429–431 (1982). 6176024 10.1126/science.6176024

[b9] Aarons I. Renal Immunofluorescence in Nzb-Nzw Mice. Nature 203, 1080–1081 (1964). 14223091 10.1038/2031080a0

[b10] Dai C. *et al.* Interferon alpha on NZM2328.Lc1R27: Enhancing autoimmunity and immune complex-mediated glomerulonephritis without end stage renal failure. Clin immunol 154, 66–71 (2014). 24981059 10.1016/j.clim.2014.06.008 PMC4167638

[b11] Jorgensen T. N., Roper E., Thurman J. M., Marrack P. & Kotzin B. L. Type I interferon signaling is involved in the spontaneous development of lupus-like disease in B6.Nba2 and (B6.Nba2 × NZW)F(1) mice. Genes Immun 8, 653–662 (2007). 17882225 10.1038/sj.gene.6364430

[b12] Rozzo S. J. *et al.* Evidence for an interferon-inducible gene, Ifi202, in the susceptibility to systemic lupus. Immunity 15, 435–443 (2001). 11567633 10.1016/s1074-7613(01)00196-0

[b13] Sriram U. *et al.* Myeloid dendritic cells from B6.NZM Sle1/Sle2/Sle3 lupus-prone mice express an IFN signature that precedes disease onset. J Immunol 189, 80–91 (2012). 22661089 10.4049/jimmunol.1101686 PMC3381850

[b14] Li J. Z. *et al.* [Study on experimental systemic lupus erythematosus mouse model induced by pristane]. Xi bao yu fen zi mian yi xue za zhi 27, 119–122 (2011). 21315035

[b15] Moisini I. *et al.* The Yaa locus and IFN-alpha fine-tune germinal center B cell selection in murine systemic lupus erythematosus. J Immunol 189, 4305–4312 (2012). 23024275 10.4049/jimmunol.1200745 PMC3478483

[b16] Hadj-Slimane R., Chelbi-Alix M. K., Tovey M. G. & Bobe P. An essential role for IFN-alpha in the overexpression of Fas ligand on MRL/lpr lymphocytes and on their spontaneous Fas-mediated cytotoxic potential. J Interferon Cytokine Res 24, 717–728 (2004). 15684739 10.1089/jir.2004.24.717

[b17] Ito T., Kanzler H., Duramad O., Cao W. & Liu Y. J. Specialization, kinetics, and repertoire of type 1 interferon responses by human plasmacytoid predendritic cells. Blood 107, 2423–2431 (2006). 16293610 10.1182/blood-2005-07-2709

[b18] Ju X., Clark G. & Hart D. N. Review of human DC subtypes. Methods Mol Biol 595, 3–20 (2010). 19941102 10.1007/978-1-60761-421-0_1

[b19] Nakano H., Yanagita M. & Gunn M. D. CD11c(+)B220(+)Gr-1(+) cells in mouse lymph nodes and spleen display characteristics of plasmacytoid dendritic cells. J Exp Med 194, 1171–1178 (2001). 11602645 10.1084/jem.194.8.1171 PMC2193516

[b20] Wang Y. H. & Liu Y. J. Mysterious origin of plasmacytoid dendritic cell precursors. Immunity 21, 1–2 (2004). 15345213 10.1016/j.immuni.2004.07.003

[b21] Means T. K. *et al.* Human lupus autoantibody-DNA complexes activate DCs through cooperation of CD32 and TLR9. J Clin Invest 115, 407–417 (2005). 15668740 10.1172/JCI23025 PMC544604

[b22] Vollmer J. *et al.* Immune stimulation mediated by autoantigen binding sites within small nuclear RNAs involves Toll-like receptors 7 and 8. J Exp Med 202, 1575–1585 (2005). 16330816 10.1084/jem.20051696 PMC2213330

[b23] Gaipl U. S. *et al.* Clearance deficiency and systemic lupus erythematosus (SLE). J Autoimmun 28, 114–121 (2007). 17368845 10.1016/j.jaut.2007.02.005

[b24] Carrington E. M. *et al.* Prosurvival Bcl-2 family members reveal a distinct apoptotic identity between conventional and plasmacytoid dendritic cells. Proc Natl Acad Sci USA 112, 4044–4049 (2015). 25775525 10.1073/pnas.1417620112 PMC4386329

[b25] Zhan Y. *et al.* Bcl-2 Antagonists Kill Plasmacytoid Dendritic Cells From Lupus-Prone Mice and Dampen Interferon-alpha Production. Arthritis Rheumatol 67, 797–808 (2015). 25418983 10.1002/art.38966

[b26] Hahn B. H. & Kono D. In Dubois’ Lupus Erythematosus and Related Syndromes (Eighth Edition). 190–236 (W.B. Saunders, 2013).

[b27] Villadangos J. A. & Young L. Antigen-presentation properties of plasmacytoid dendritic cells. Immunity 29, 352–361 (2008). 18799143 10.1016/j.immuni.2008.09.002

[b28] Contractor N., Louten J., Kim L., Biron C. A. & Kelsall B. L. Cutting edge: Peyer’s patch plasmacytoid dendritic cells (pDCs) produce low levels of type I interferons: possible role for IL-10, TGFbeta, and prostaglandin E2 in conditioning a unique mucosal pDC phenotype. J Immunol 179, 2690–2694 (2007). 17709480 10.4049/jimmunol.179.5.2690

[b29] Bjorck P., Leong H. X. & Engleman E. G. Plasmacytoid dendritic cell dichotomy: identification of IFN-alpha producing cells as a phenotypically and functionally distinct subset. J Immunol 186, 1477–1485 (2011). 21172865 10.4049/jimmunol.1000454 PMC3138736

[b30] Kadowaki N. *et al.* Subsets of human dendritic cell precursors express different toll-like receptors and respond to different microbial antigens. J Exp Med 194, 863–869 (2001). 11561001 10.1084/jem.194.6.863 PMC2195968

[b31] Gleisner M. A. *et al.* Dendritic and stromal cells from the spleen of lupic mice present phenotypic and functional abnormalities. Mol Immunol 54, 423–434 (2013). 23428837 10.1016/j.molimm.2013.01.011

[b32] Andrews B. S. *et al.* Spontaneous murine lupus-like syndromes. Clinical and immunopathological manifestations in several strains. J Exp Med 148, 1198–1215 (1978). 309911 10.1084/jem.148.5.1198 PMC2185049

[b33] Fiore N. *et al.* Immature myeloid and plasmacytoid dendritic cells infiltrate renal tubulointerstitium in patients with lupus nephritis. Mol Immunol 45, 259–265 (2008). 17570528 10.1016/j.molimm.2007.04.029

[b34] Furukawa F. & Yoshimasu T. Animal models of spontaneous and drug-induced cutaneous lupus erythematosus. Autoimmun Rev 4, 345–350 (2005). 16081025 10.1016/j.autrev.2005.01.006

[b35] Furukawa F. Photosensitivity in cutaneous lupus erythematosus: lessons from mice and men. J Dermatol Sci 33, 81–89 (2003). 14581133 10.1016/j.jdermsci.2003.08.005

[b36] Perry D., Sang A., Yin Y., Zheng Y. Y. & Morel L. Murine models of systemic lupus erythematosus. J Biomed Biotechnol 2011, 271694 (2011). 21403825 10.1155/2011/271694 PMC3042628

[b37] Murphy E. D. & Roths J. B. A Y chromosome associated factor in strain BXSB producing accelerated autoimmunity and lymphoproliferation. Arthritis Rheum 22, 1188–1194 (1979). 315777 10.1002/art.1780221105

[b38] Subramanian S. *et al.* A Tlr7 translocation accelerates systemic autoimmunity in murine lupus. Proc Natl Acad Sci USA 103, 9970–9975 (2006). 16777955 10.1073/pnas.0603912103 PMC1502563

[b39] Pisitkun P. *et al.* Autoreactive B cell responses to RNA-related antigens due to TLR7 gene duplication. Science 312, 1669–1672 (2006). 16709748 10.1126/science.1124978

[b40] Haywood M. E. *et al.* Dissection of BXSB lupus phenotype using mice congenic for chromosome 1 demonstrates that separate intervals direct different aspects of disease. J Immunol 173, 4277–4285 (2004). 15383556 10.4049/jimmunol.173.7.4277

[b41] Santiago-Raber M. L. *et al.* Evidence for genes in addition to Tlr7 in the Yaa translocation linked with acceleration of systemic lupus erythematosus. J Immunol 181, 1556–1562 (2008). 18606711 10.4049/jimmunol.181.2.1556

[b42] Deane J. A. *et al.* Control of toll-like receptor 7 expression is essential to restrict autoimmunity and dendritic cell proliferation. Immunity 27, 801–810 (2007). 17997333 10.1016/j.immuni.2007.09.009 PMC2706502

[b43] Rowland S. L. *et al.* Early, transient depletion of plasmacytoid dendritic cells ameliorates autoimmunity in a lupus model. J Exp Med 211, 1977–1991 (2014). 25180065 10.1084/jem.20132620 PMC4172228

[b44] Davison L. M. & Jorgensen T. N. Sialic Acid-Binding Immunoglobulin-Type Lectin H-Positive Plasmacytoid Dendritic Cells Drive Spontaneous Lupus-like Disease Development in B6.Nba2 Mice. Arthritis Rheumatol 67, 1012–1022 (2015). 25504931 10.1002/art.38989

[b45] Nickerson K. M., Cullen J. L., Kashgarian M. & Shlomchik M. J. Exacerbated autoimmunity in the absence of TLR9 in MRL.Fas(lpr) mice depends on Ifnar1. J Immunol 190, 3889–3894 (2013). 23467932 10.4049/jimmunol.1203525 PMC3622185

[b46] Yu P. *et al.* Toll-like receptor 9-independent aggravation of glomerulonephritis in a novel model of SLE. Int Immunol 18, 1211–1219 (2006). 16798839 10.1093/intimm/dxl067

[b47] Christensen S. R. *et al.* Toll-like receptor 7 and TLR9 dictate autoantibody specificity and have opposing inflammatory and regulatory roles in a murine model of lupus. Immunity 25, 417–428 (2006). 16973389 10.1016/j.immuni.2006.07.013

[b48] Santiago-Raber M. L. *et al.* Critical role of TLR7 in the acceleration of systemic lupus erythematosus in TLR9-deficient mice. J Autoimmun 34, 339–348 (2010). 19944565 10.1016/j.jaut.2009.11.001

[b49] Nickerson K. M. *et al.* TLR9 regulates TLR7- and MyD88-dependent autoantibody production and disease in a murine model of lupus. J Immunol 184, 1840–1848 (2010). 20089701 10.4049/jimmunol.0902592 PMC4098568

[b50] Vremec D., Pooley J., Hochrein H., Wu L. & Shortman K. CD4 and CD8 expression by dendritic cell subtypes in mouse thymus and spleen. J Immunol 164, 2978–2986 (2000). 10706685 10.4049/jimmunol.164.6.2978

