# Supplementary material for: Phenotypic and functional alterations of pDCs in lupus-prone mice
Source: Sci Rep. 2016 Feb 16;6:20373. doi: 10.1038/srep20373 (PMC4754657; doi:10.1038/srep20373)

Phenotypic and functional alterations of pDCs in lupus-prone mice

Zhenyuan Zhou[1], Jianyang Ma[1], Chunyuan Xiao[1], Xiao Han[2], Rong Qiu[2], Yan Wang[2], Yingying Zhou[1], Xinfang Huang[1, *] and Nan Shen[1&2, 3*]

1. Department of Rheumatology, Renji Hospital, Shanghai Jiao Tong university School of Medicine, Shanghai, China
2. Institute of Health Sciences, Shanghai Institutes for Biological Sciences (SIBS) & Shanghai Jiao Tong University School of Medicine (SJTUSM), Chinese Academy of Sciences (CAS), Shanghai, China
3. Division of Rheumatology and the Center for Autoimmune Genomics and Etiology (CAGE), Cincinnati Children's Hospital Medical Center, Cincinnati, Ohio, United States of America

Corresponding to: Dr. Xingfang Huang, hxf343@126.com
Dr. Nan Shen, nanshensibs@gmail.com

Supplementary materials

Supplementary Table 1: Cell counts at pre-lupus stage (×10^6)

Strains	Spleen	Bone Marrow**	Brachial Lymph node	Thymus
NZB/W F1	109.9±8.1	42.1±2.1	2.8±0.7	136.7±8.2
NZB	92.0±5.1	39.1±5.7	1.7±0.4	148.6±10.7
NZW	98.4±6.4	43.4±5.7	1.7±0.4	121±8.5
NZM2410	107.3±6.5	41.9±4.3	5.3±1.5	101.6±8.3
MRL-*lpr*	109.2±4.0	43.3±4.0	2.1±0.5	154.3±12.8
B6.NZM$^{Sle1/2/3}$	106.7±5.5	43.0±6.3	1.8±0.3	104.1±7.1
BXSB/Mp	105.8±6.7	48.3±5.8	1.7±0.4	142.4±8.4
C57BL/6	93.4±5.1	47.3±6.1	1.7±0.3	134.1±6.3

**Bone marrow: Total cells from 2 femur and 2 tibias.

Supplementary Table 2: LN pDC and renal pDC counts at different disease stage in different lupus prone strains.

	Branchial LN pDC (Mean±SD)×10^3			Renal pDC (Mean±SD)×10^3		
Strains	Pre-lupus	Early-lupus	Advanced lupus	Pre-lupus	Early-lupus	Advanced lupus
NZB/W F1	25.25±2.73	25.97±3.63	22.69±2.98	ND	9.65±8.91	ND
MRL-lpr	26.50	26.50±3.16	39.73±7.91*	ND	8.61±6.70	ND
B6.NZMSle1/2/3	24.50	24.50±2.79	24.50±2.79	ND	5.30±5.90	ND
C57BL/6	27.10	26.25±3.83	27.60±4.01	ND	ND	ND

*p<0.05

Supplementary Table 3： Primers for qPCR

Genes	Forward primer	Reverse primer
Mouse GAPDH	ACCACAGTCCATGCCATCAC	CACCACCCTGTTGCTGTAGCC
Mouse IFNA	GGACTTTGGATTCCCGCAGGAGAAG	GCTGCATCAGACAGCCTTGCAGGTC
Mouse MX1	GATCCGACTTCACTTCCAGATGG	CATCTCAGTGGTAGTCAACCC
Mouse IFIT2	GGAAAAAGAAAGCCCTCACC	GTTCCCCAAACTCCTGACAA
Mouse CXCL10	GGATGGCTGTCCTAGCTCTG	TGAGCTAGGGAGGACAAGGA

Supplementary Figure 1: pDC purity after Nycodenz enrichment and after sorting. Sample data were spleen cells from C57BL/6 mice.

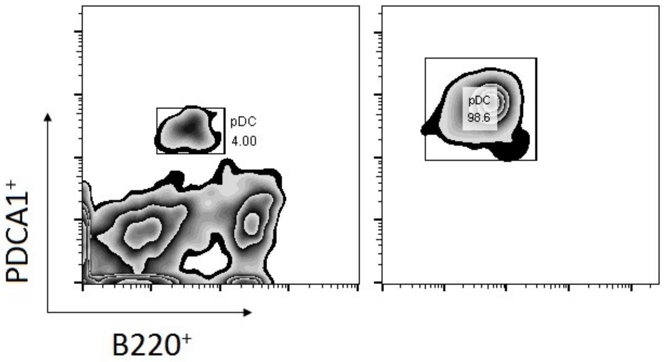

Supplementary Figure 2: Gating Strategy: Sample data were pDCs from C57BL/6 mice. A) pDC gating strategies of spleen pDC. B) Dead cell staining of purified pDC before and after stimulation.

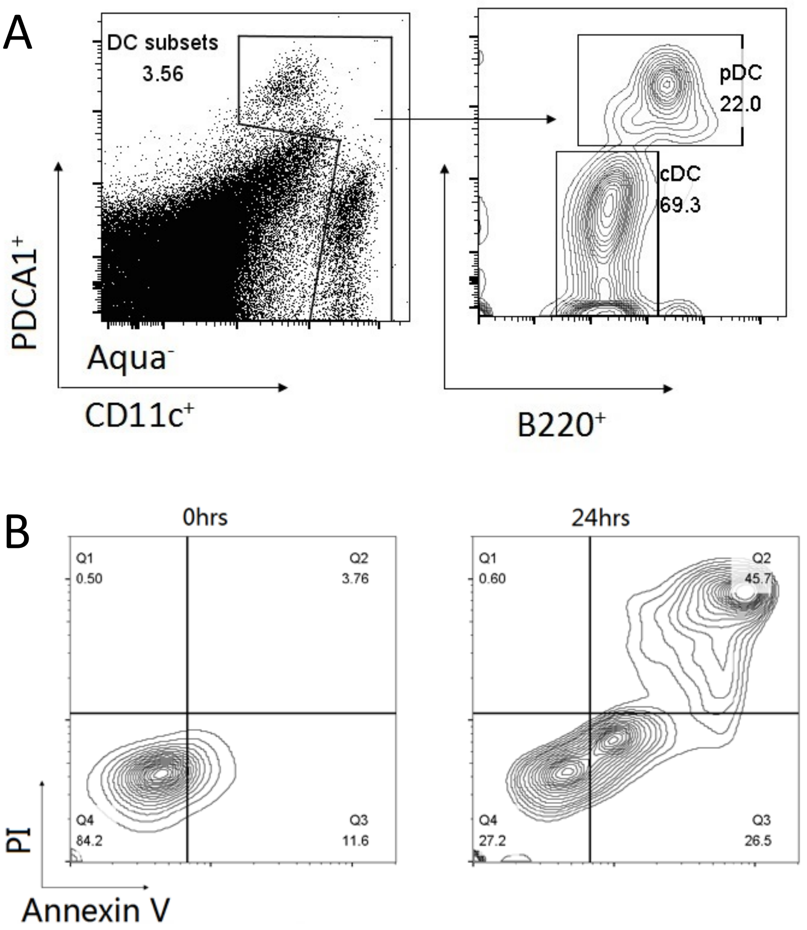

Supplementary Figure 3:

A) FAC results of renal pDC after Nycodenz enrichment: The mouse kidney were cutting into small pieces and then digested with 0.1mg/mL collagenase for 20min at room temperature. After digestion, total cell were collected and filtered through 40μM strainer.

pDC were enriched by using 1.077g/ml Nycodenz. The sample data was NZB/W F1 mice at different disease stage. The actual pDC numbers in kidney were extreme low. B) The immunofluorescent microscopy sample data of kidney tissue from one 30-week-old NZB/W F1 mice. Neither CD11c cell nor B220 cell could be directly detected in immunoflourescent microscopy.

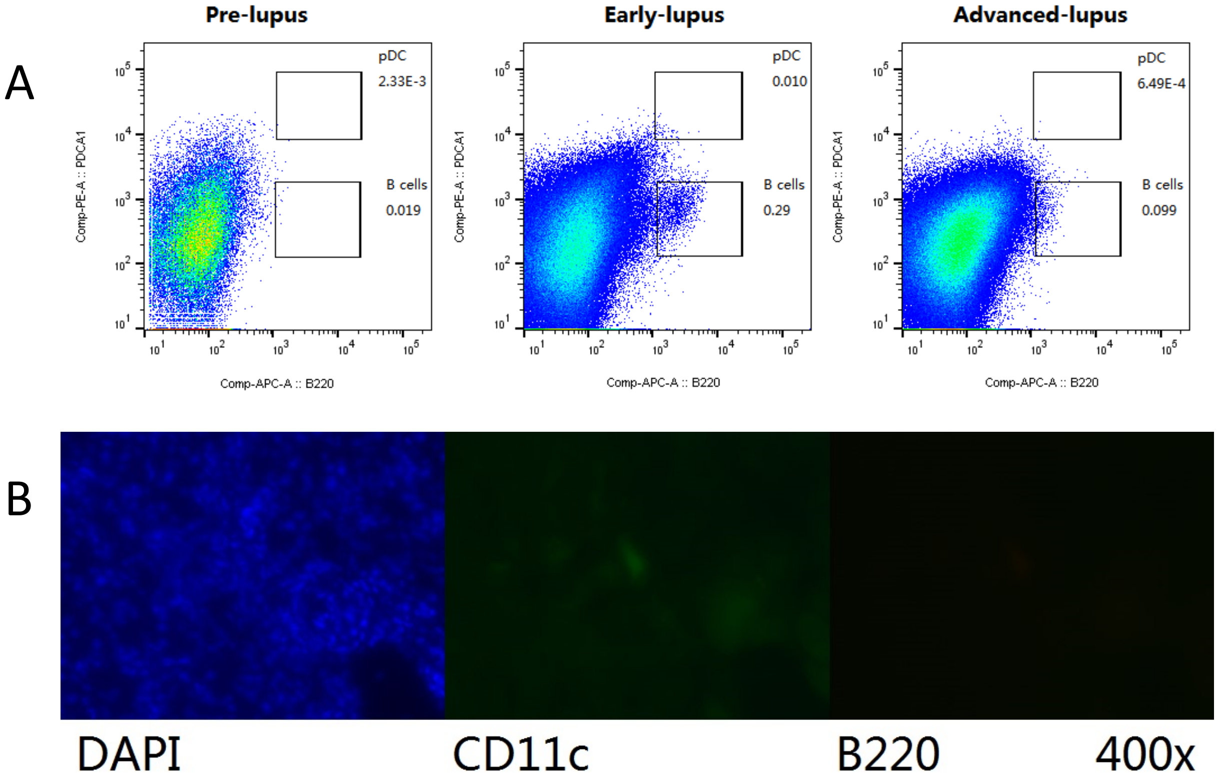

Supplement: Supplementary Information [file srep20373-s1.pdf]
